# Correction: Seasonal changes in diet and chemical defense in the Climbing Mantella frog (*Mantella laevigata*)

**DOI:** 10.1371/journal.pone.0218981

**Published:** 2019-06-20

**Authors:** The PLOS ONE Staff

Hadley Weiss is not included in the author byline. Hadley Weiss should be listed as the 25th author and affiliated with LS50: Integrated Science Freshman Class, Harvard University, Cambridge, Massachusetts, United States of America. The contributions of this author are as follows: Investigation and Writing–Review & Editing.

The publisher apologizes for the error.
