# Supplementary figures and images for: Seasonal changes in diet and chemical defense in the Climbing Mantella frog (Mantella laevigata)
Source: PLoS One. 2018 Dec 26;13(12):e0207940. doi: 10.1371/journal.pone.0207940 (PMC6306172; doi:10.1371/journal.pone.0207940)

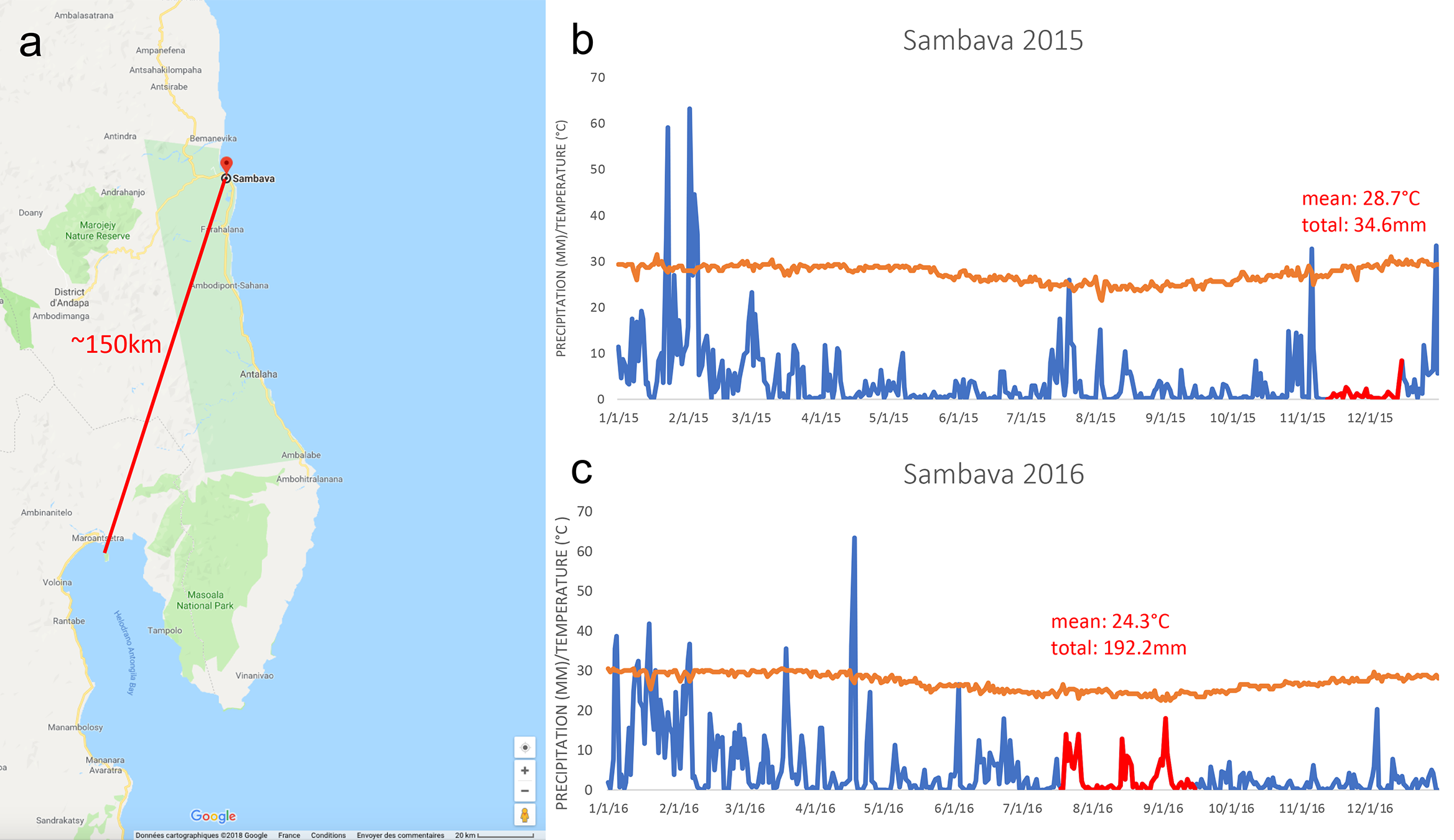

Supplement: S1 Fig — (a) Sambava is roughly 150 km northeast of Nosy Mangabe. Precipitation (blue) and temperature (orange) are shown for 2015 (b) and 2016 (c); field work time is indicated in red. Environmental data was obtained from: https://www.historique-meteo.net/afrique/madagascar/sambava/ (TIF) [file pone.0207940.s001.tif]
