# Supplementary material for: Seasonal changes in diet and chemical defense in the Climbing Mantella frog (Mantella laevigata)
Source: PLoS One. 2018 Dec 26;13(12):e0207940. doi: 10.1371/journal.pone.0207940 (PMC6306172; doi:10.1371/journal.pone.0207940)
Supplement: S2 Table — The details for each cytochrome oxidase 1 barcoded non-ant arthropod is listed with the sample ID, the prey type, and information on the top BLAST hit including the species name, percent identity, and E value. (DOCX) [file pone.0207940.s003.docx]

| Sample ID | Category | GenBank Accession | BLASTn match | BLASTn match GenBank Accession | Similarity | E Value |
| --- | --- | --- | --- | --- | --- | --- |
| 7007-060 | Beetle | MG947255 | *Trixagus carinicollis* | KR488365 | 79% | 1.53E-156 |
| 7010-003 | Beetle | MG947290 | *Lagria atripes* | KX505309 | 89% | 0 |
| 7011-001 | Beetle | MG947298 | *Idolus picipennis* | KM441319 | 87% | 0 |
| 7502-018 | Beetle | MG947316 | *Liroetis* sp. | KC185724 | 87% | 0 |
| 7506-009 | Beetle | MG947358 | *Dalopius exilis* | KM612529 | 86% | 0 |
| 7509-009 | Beetle | MG947379 | *Crossotarsus externedentatus* | KR261316 | 99.80% | 0 |
| 7505-002 | Beetlr | MG947352 | *Stricticollis valgipes* | KU188427 | 84% | 0 |
| 7505-035 | Booklice | MG947355 | Psocoptera | HQ978916 | 83% | 0 |
| 7505-045 | Booklice | MG947356 | Psocoptera | HQ978916 | 83% | 0 |
| 7500-001 | Butterfly/Moth | MG947299 | Lepidoptera | GU698872 | 91% | 0 |
| 7502-019 | Fly | MG947317 | Ceratopogonidae | KJ164150 | 88% | 0 |
| 7508-001 | Fly | MG947372 | *Chrysomya villeneuvi* | KX096339 | 95% | 0 |
| 7508-007 | Fly | MG947374 | *Drosophila malerkotliana* | JQ679118 | 89% | 0 |
| 7508-008 | Fly | MG947375 | *Drosophila fengkainensis* | AB669754 | 89% | 0 |
| 7508-013 | Fly | MG947378 | *Dohrniphora cornuta* | KR666968 | 99.10% | 0 |
| 7007-046 | Gnat | MG947249 | Sciaridae sp. | KM955768 | 90% | 0 |
| 7502-064 | Gnat | MG947331 | Sciaridae | KR474859 | 88% | 0 |
| 7504-002 | Midge | MG947336 | *Forcipomyia* sp. | KR440222 | 90% | 0 |
| 7504-004 | Midge | MG947337 | *Forcipomyia* sp. | KR440222 | 90% | 0 |
| 7504-006 | Midge | MG947338 | *Forcipomyia* sp. | KR440223 | 90% | 0 |
| 7504-023 | Midge | MG947343 | *Forcipomyia* sp. | KR440222 | 90% | 0 |
| 7504-035 | Midge | MG947345 | *Forcipomyia* sp. | KR432061 | 90% | 0 |
| 7504-039 | Midge | MG947346 | *Forcipomyia* sp. | KR432061 | 90% | 0 |
| 7504-047 | Midge | MG947347 | *Forcipomyia* sp. | KR432061 | 90% | 0 |
| 7505-025 | Midge | MG947354 | *Forcipomyia* sp. | KJ730007 | 86% | 0 |
| 7508-004 | Midge | MG947373 | *Forcipomyia* sp. | KR432061 | 90% | 0 |
| 7508-009 | Midge | MG947376 | *Forcipomyia sp.* | KR432061 | 90% | 0 |
| 7003-049 | Mite | MG947246 | Galumnidae | KM832007 | 81% | 1.50E-137 |
| 7010-010 | Mite | MG947291 | Scheloribatidae | KR070270 | 82% | 5.50E-180 |
| 7010-015 | Mite | MG947292 | Galumnidae | KM838551 | 80% | 2.50E-146 |
| 7010-016 | Mite | MG947293 | Galumnidae | KM832007 | 81% | 9.97E-139 |
| 7010-020 | Mite | MG947294 | Suctobelbidae | KM834212 | 80% | 9.96E-158 |
| 7010-021 | Mite | MG947295 | Galumnidae | KM832007 | 81% | 3.97E-131 |
| 7010-023 | Mite | MG947296 | Galumnidae | KM830795 | 80% | 4.23E-156 |
| 7500-003 | Mite | MG947300 | Ologamasidae | KM832156 | 81% | 1.26E-176 |
| 7502-035 | Mite | MG947322 | Ceratozetidae sp. | KM824002 | 81% | 1.84E-174 |
| 7503-001 | Mite | MG947333 | Oppiidae | JX833797 | 81% | 1.52E-175 |
| 7503-035 | Mite | MG947334 | Scheloribatidae | KR070270 | 80% | 7.86E-173 |
| 7503-045 | Mite | MG947335 | *Eueremaeus* sp. | KM835787 | 81% | 1.85E-155 |
| 7504-007 | Mite | MG947339 | Ceratozetidae | KM824002 | 80% | 2.25E-173 |
| 7504-094 | Mite | MG947348 | *Berniniella hauseri* | KF293512 | 83% | 0 |
| 7504-095 | Mite | MG947349 | Sarcoptiformes sp. | KM837350 | 79% | 4.21E-144 |
| 7504-097 | Mite | MG947350 | *Scutovertex sculptus* | GQ890430 | 80% | 1.57E-168 |
| 7504-098 | Mite | MG947351 | Ceratozetidae | KM828928 | 80% | 1.29E-150 |
| 7505-006 | Mite | MG947353 | Scheloribatidae | KR070270 | 82% | 0 |
| 7507-011 | Mosquito | MG947368 | *Anopheles hinesorum* | JX219734 | 88% | 0 |
| 7508-010 | Mosquito | MG947377 | *Anopheles albitarsis* | KM391805 | 88% | 0 |
| 7502-021 | Moth | MG947319 | *Cryptophasa albacosta* | KF395897 | 85% | 0 |
| 7507-010 | Moth | MG947367 | *Herpetogramma basalis* | HQ953218 | 93% | 0 |
| 7507-024 | Moth | MG947370 | *Herpetogramma basalis* | HQ953218 | 93% | 0 |
| 7502-002 | Spider | MG947305 | *Neospintharus trigonum* | GU682677 | 89% | 0.00E+00 |
| 7502-046 | Spider | MG947324 | *Micrathena gracilis* | KY129671 | 84% | 0 |
| 7502-050 | Spider | MG947326 | Mysmeninae | GU456882 | 99% | 0 |
| 7504-010 | Spider | MG947340 | *Octonoba yesoensis* | JN817081 | 90% | 0 |
| 7501-001 | Springtail | MG947302 | *Entomobrya clitellaria* | KM610068 | 81% | 1.03E-177 |
| 7507-022 | Springtail | MG947369 | *Entomobrya clitellaria* | KM610068 | 80% | 1.53E-175 |
| 7507-025 | Springtail | MG947371 | *Homidia socia* | KJ781784 | 83% | 0 |
| 7502-008 | Termite | MG947309 | *Nasutitermes triodiae* | JX144940 | 90% | 0 |
| 7502-065 | Tick | MG947332 | Phenopelopidae | KM827886 | 81% | 0 |
| 7502-033 | Wasp | MG947321 | *Probaryconus* sp. | JX303731 | 80% | 2.73E-172 |
| 7502-047 | Wasp | MG947325 | *Dinotrema* sp. | KR792708 | 93% | 0 |
| 7502-054 | Wasp | MG947328 | Alysiinae | JX828624 | 91% | 0 |
| 7502-058 | Wasp | MG947329 | *Asobara citri* | JQ808423 | 92% | 0 |
| 7502-061 | Wasp | MG947330 | *Asobara citri* | JQ808423 | 92% | 0 |
| 7504-030 | Weevil | MG947344 | Platypodinae | KT696208 | 100% | 0 |
