# Supplementary material for: Seasonal changes in diet and chemical defense in the Climbing Mantella frog (Mantella laevigata)
Source: PLoS One. 2018 Dec 26;13(12):e0207940. doi: 10.1371/journal.pone.0207940 (PMC6306172; doi:10.1371/journal.pone.0207940)
Supplement: S5 Table — Both morphologically and genetically identifiable ant specimens with exemplar photos representing respective taxonomic groups across seasonal groups are listed with their sample ID’s. (DOCX) [file pone.0207940.s006.docx]

| Sample ID | Seasonal group | Genus | # with BLASTn match to Genus | Scale bar size (mm) | Specimen photo |
| --- | --- | --- | --- | --- | --- |
| 7502-006 | Wet | *Tapinoma* | 1 | 1.0 | 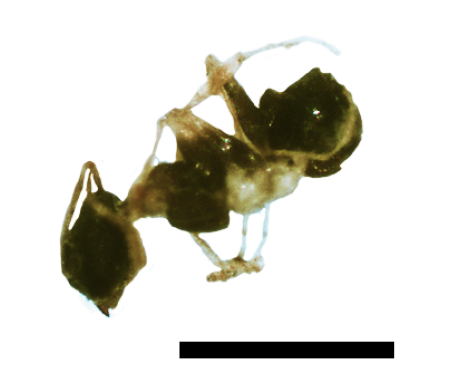 |
| 7007-074 | Dry | *Pheidole* | 92 | 1.0 | 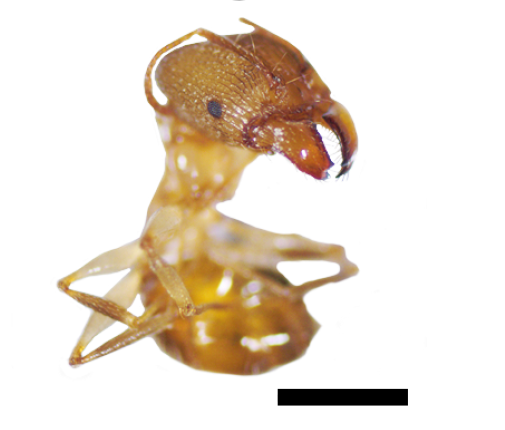 |
| 7007-102 | Dry | *Pheidole* | 92 | 1.0 | 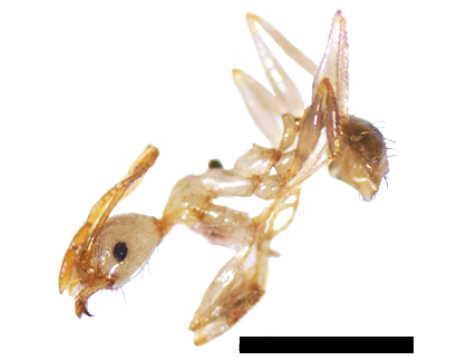 |
| 7007-081 | Dry | *Pheidole* | 92 | 1.0 | 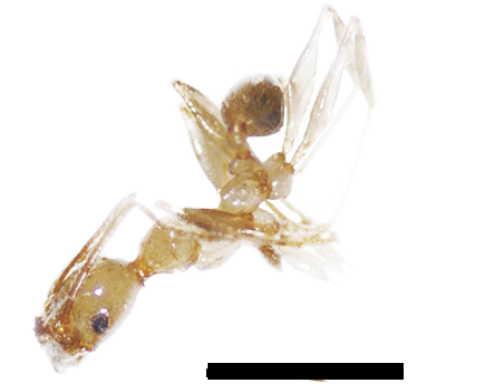 |
| 7003-045 | Dry | *Pheidole* | 92 | 1.0 | 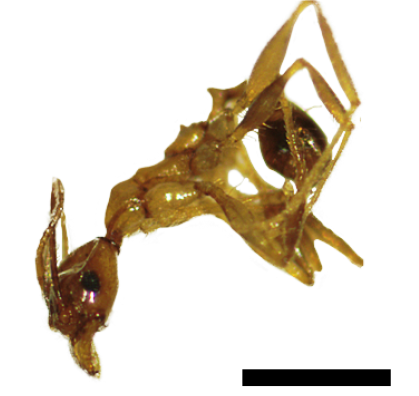 |
| 7003-006 | Dry | *Pheidole* | 92 | 1.0 | 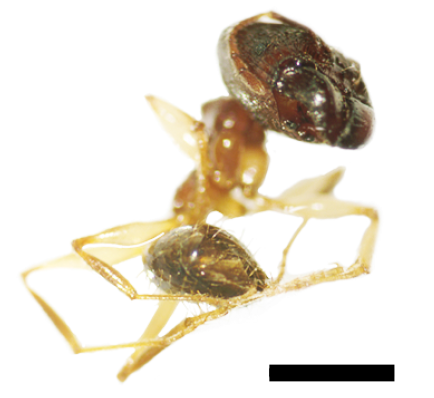 |
| 7003-048 | Dry | *Paratrechina* | 1 | 1.0 | 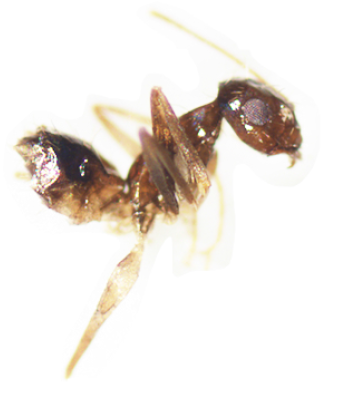 |
| 7003-037 | Dry | *Strumigenys* | 1 | 1.0 | 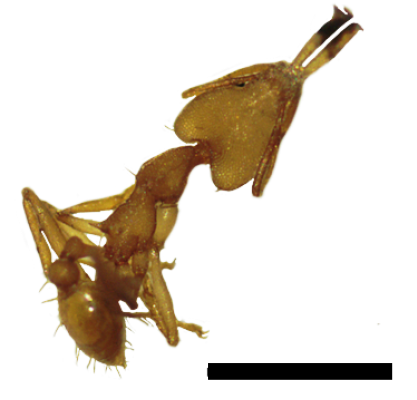 |
| 7009-022 | Dry | *Pheidole* | 92 | 1.0 | 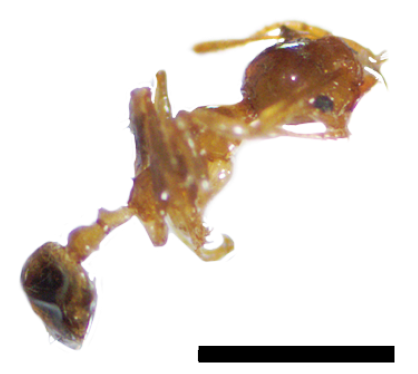 |
| 7009-029 | Dry | *Pheidole* | 92 | 1.0 | 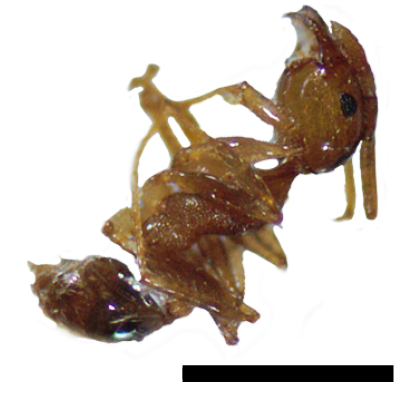 |
| 7502-016 | Wet | *Tetramorium* | 3 | 1.0 | 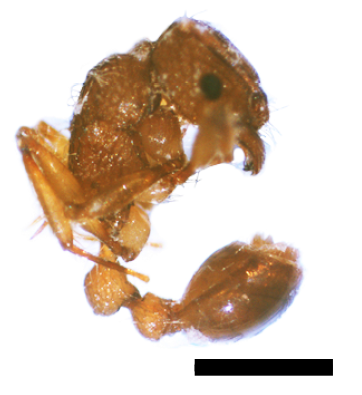 |
| 7502-004 | Wet | *Pheidole* | 92 | 1.0 | 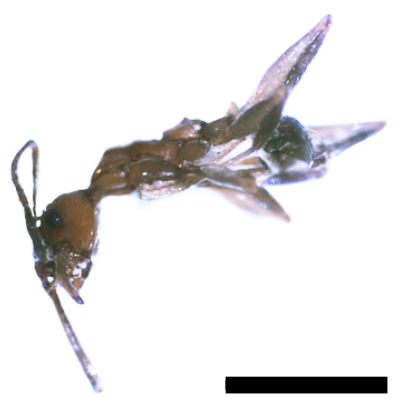 |
| 7003-026 | Dry | *Pheidole* | 92 | 1.0 | 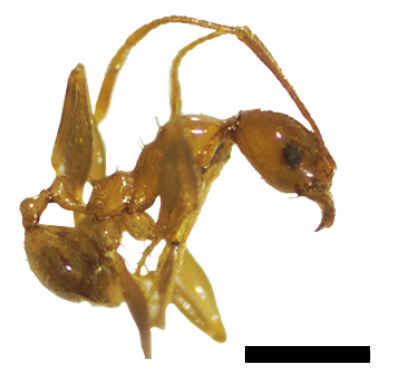 |
