# Supplementary material for: Seasonal changes in diet and chemical defense in the Climbing Mantella frog (Mantella laevigata)
Source: PLoS One. 2018 Dec 26;13(12):e0207940. doi: 10.1371/journal.pone.0207940 (PMC6306172; doi:10.1371/journal.pone.0207940)
Supplement: S6 Table — Genetically identifiable arachnid specimens with exemplar photos representing respective taxonomic groups across seasonal groups are listed with their sample ID’s. (DOCX) [file pone.0207940.s007.docx]

| Sample ID | Seasonal group | Order | # with BLASTn match to order | Scale bar size (mm) | Specimen photo |
| --- | --- | --- | --- | --- | --- |
| 7502-065 | Wet | Sarcoptiformes | 18 | 0.25 | 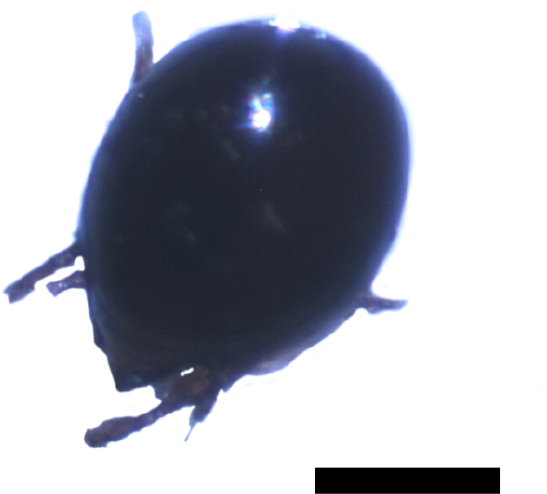 |
| 7503-035 | Wet | Sarcoptiformes | 18 | 0.25 | 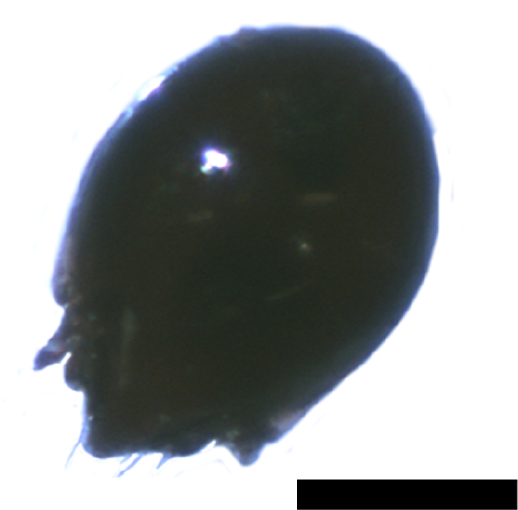 |
| 7010-010 | Dry | Sarcoptiformes | 18 | 0.25 | 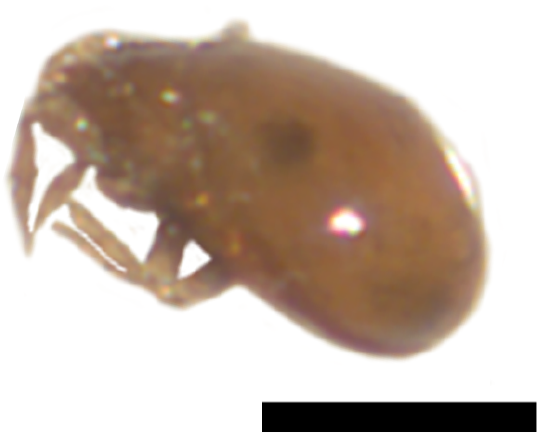 |
| 7010-015 | Dry | Sarcoptiformes | 18 | 0.25 | 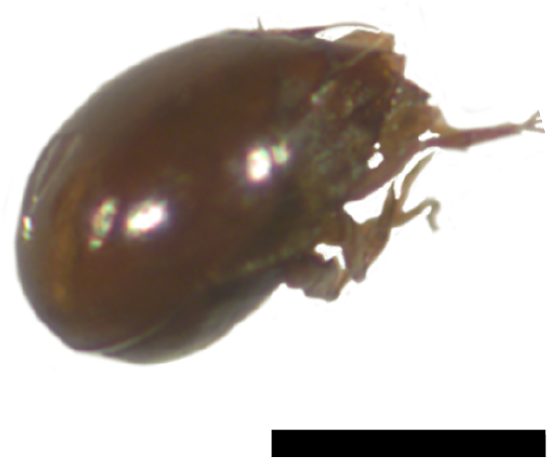 |
| 7003-049 | Dry | Sarcoptiformes | 18 | 0.25 | 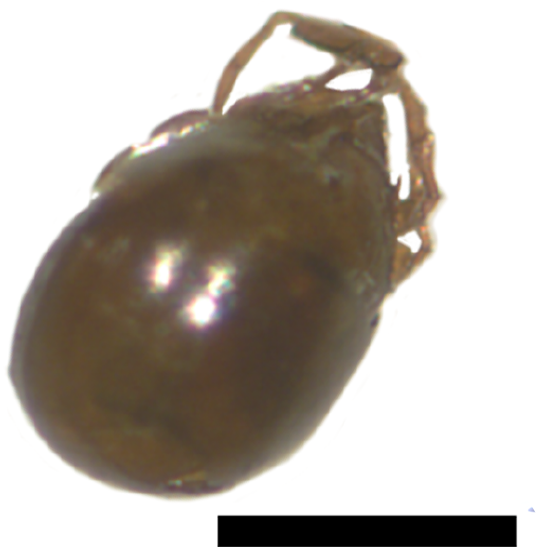 |
| 7502-050 | Wet | Araneae | 4 | 0.5 | 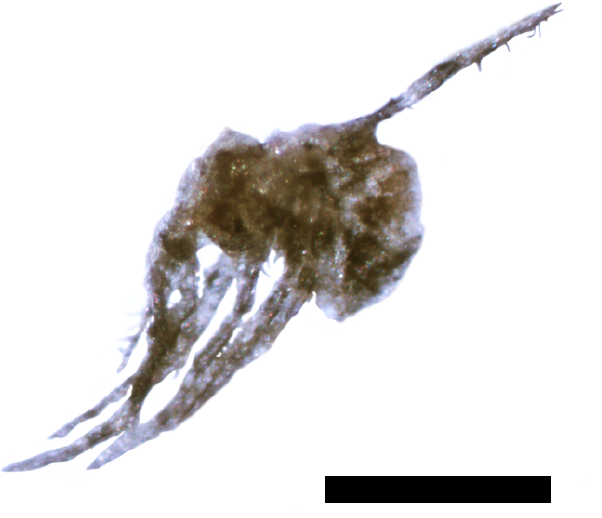 |
| 7502-002 | Wet | Araneae | 4 | 0.5 | 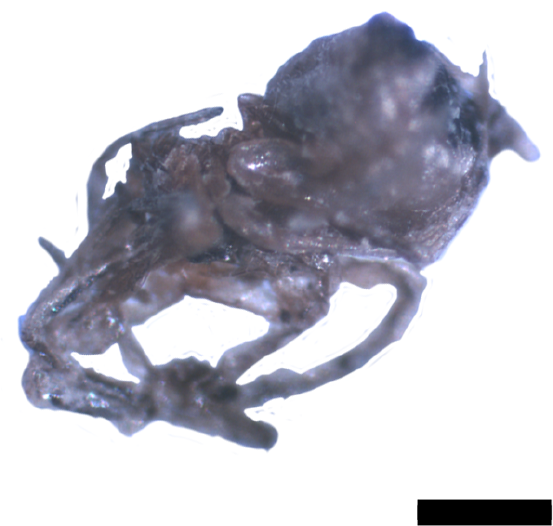 |
| 7504-010 | Wet | Araneae | 4 | 0.5 | 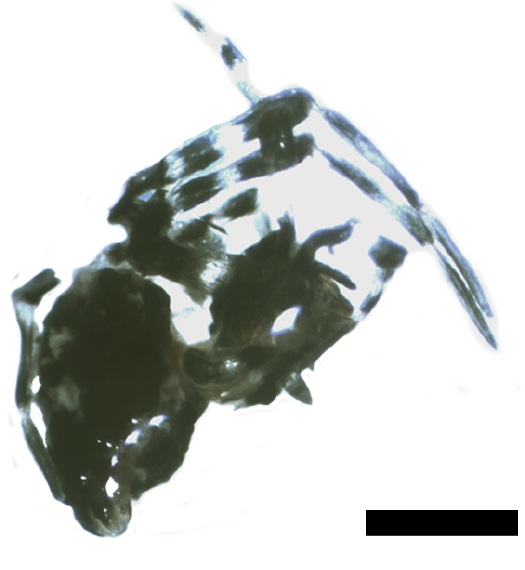 |
| 7500-003 | Wet | Mesostigmata | 1 | 0.25 | 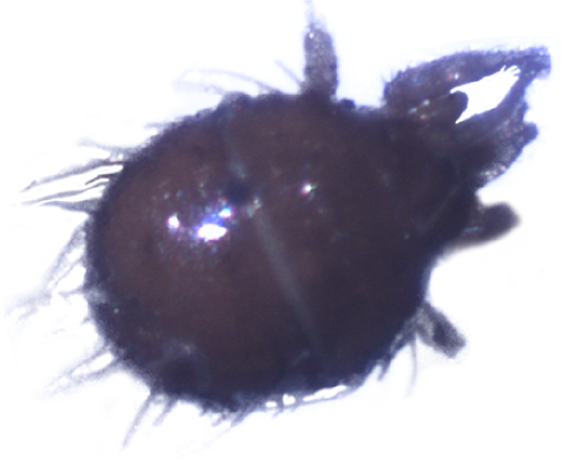 |
| 7504-095 | Wet | Sarcoptiformes | 18 | 0.25 | 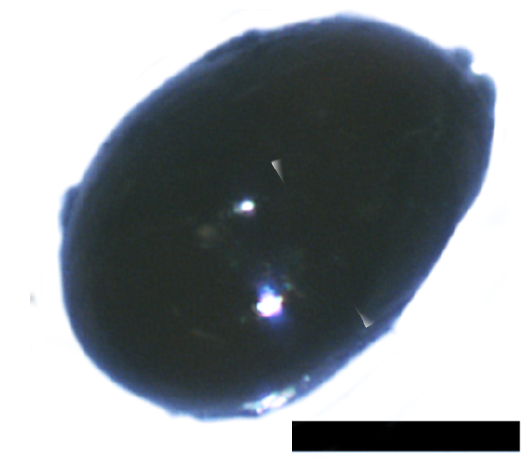 |
| 7504-098 | Wet | Sarcoptiformes | 18 | 0.25 | 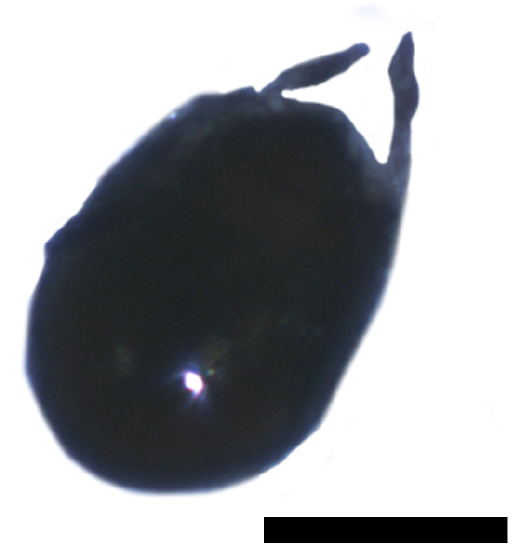 |
| 7503-001 | Wet | Sarcoptiformes | 18 | 0.25 | 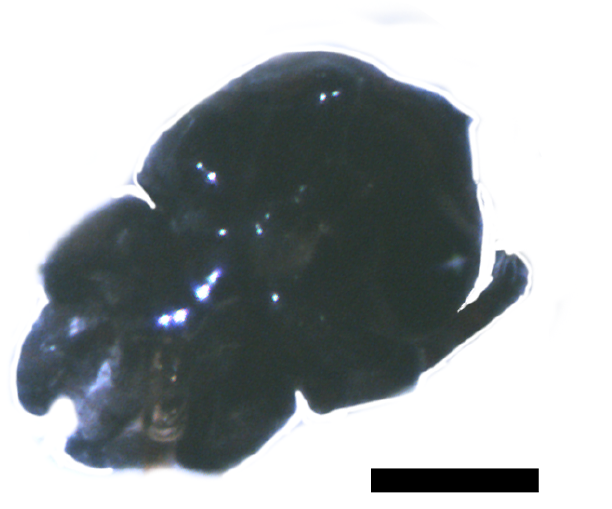 |
