# Supplementary material for: Seasonal changes in diet and chemical defense in the Climbing Mantella frog (Mantella laevigata)
Source: PLoS One. 2018 Dec 26;13(12):e0207940. doi: 10.1371/journal.pone.0207940 (PMC6306172; doi:10.1371/journal.pone.0207940)
Supplement: S7 Table — Genetically identifiable ant specimens with exemplar photos representing respective taxonomic groups across seasonal groups are listed with their sample ID’s. (DOCX) [file pone.0207940.s008.docx]

| Sample ID | Seasonal group | Order | # with BLASTn match to order | Scale bar size (mm) | Specimen photo |
| --- | --- | --- | --- | --- | --- |
| 7501-001 | Wet | Collembola | 4 | 1.0 | 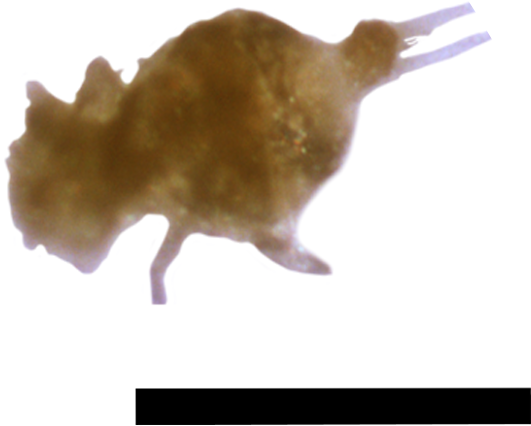 |
| 7502-018 | Wet | Coleoptera | 8 | 1.0 | 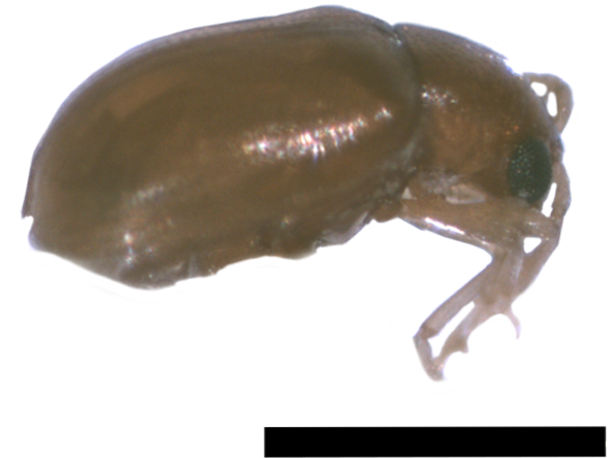 |
| 7011-001 | Dry | Coleoptera | 8 | 1.0 | 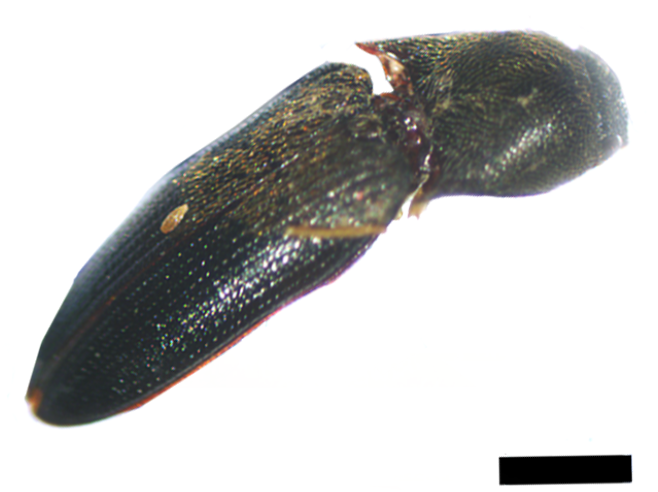 |
| 7010-003 | Dry | Coleoptera | 8 | 1.0 | 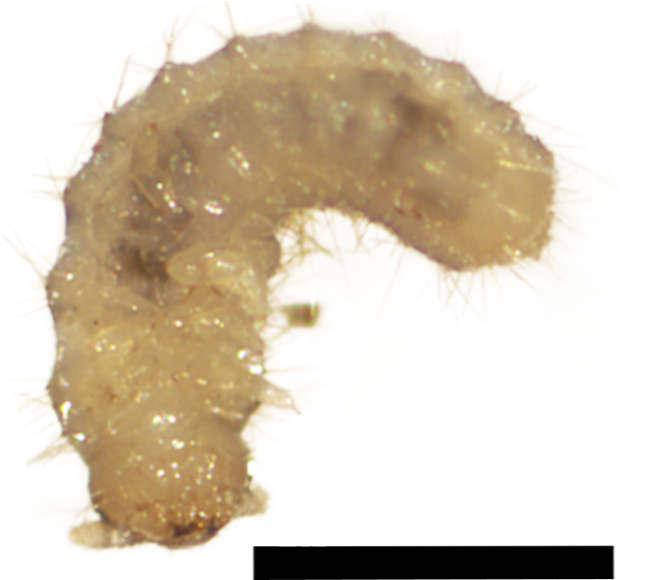 |
| 7504-047 | Wet | Diptera | 18 | 1.0 | 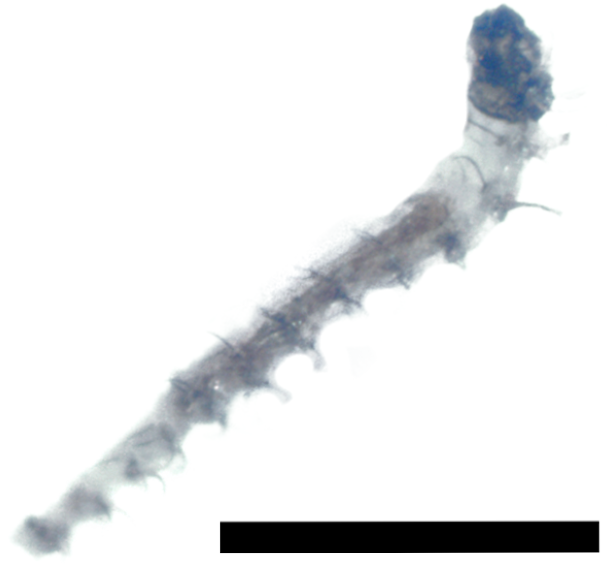 |
| 7502-058 | Wet | Hymenoptera | 7 | 1.0 | 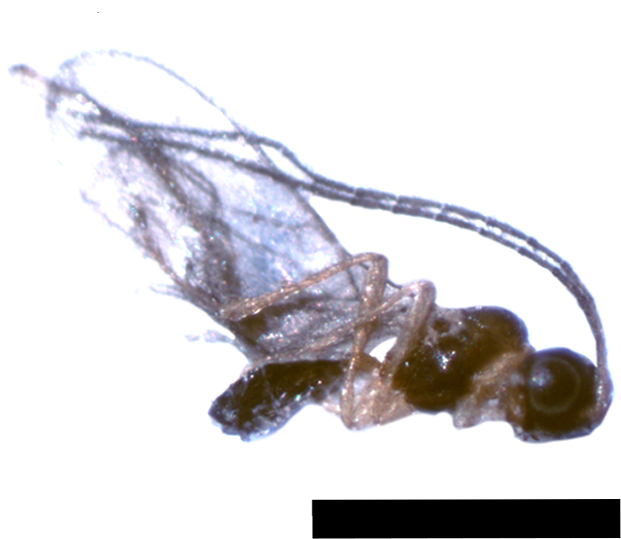 |
| 7502-061 | Wet | Hymenoptera | 7 | 1.0 | 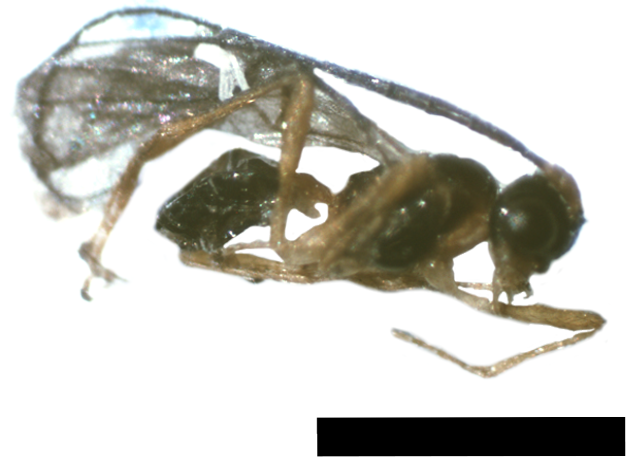 |
