# Supplementary material for: Seasonal changes in diet and chemical defense in the Climbing Mantella frog (Mantella laevigata)
Source: PLoS One. 2018 Dec 26;13(12):e0207940. doi: 10.1371/journal.pone.0207940 (PMC6306172; doi:10.1371/journal.pone.0207940)
Supplement: S8 Table — For each of the 41 identified alkaloids, we list the proposed arthropod origin and whether the alkaloid has been previously found in other mantellid species or other anurans. (DOCX) [file pone.0207940.s009.docx]

| **Alkaloid** | **Proposed arthropod origin** | **Found in Mantella (Mantellidae)** | **Found in other Anurans** | **References** |
| --- | --- | --- | --- | --- |
| 153B Unclass | Millipedes/Beetles/Unknown | No | Yes; Dendrobatidae (Oophaga) | Saporito 2007, Saporito et al 2007, Daly et al 2005, "Alkaloids:  Chemical and Biological Perspectives" 1999 |
| 189 THQ | Unknown | Yes; betsileo | No | Daly et al 2005, "Alkaloids:  Chemical and Biological Perspectives" 1999, Daly et al 1996 |
| 193D OHQ | Unknown | Yes; betsileo, laevigata, pulchra | Yes; Dendrobatidae | Daly et al 2005, "Alkaloids:  Chemical and Biological Perspectives" 1999, Daly et al 1996 |
| 195A DHQ | Myrmicine ant | Yes; betsileo, crocea, laevigata, pulchra | Yes; Dendrobatidae (Oophaga) Bufonidae, Myobatrachidae | Daly et al 2005, "Alkaloids:  Chemical and Biological Perspectives" 1999, Daly et al 1996 |
| 195J DHQ | Myrmicine ant; Solenopsis | Yes; betsileo | Yes; Dendrobatidae (Oophaga) | Daly et al 2005, "Alkaloids:  Chemical and Biological Perspectives" 1999, Jones 1999 Saporito et al 2012 |
| 209A DHQ | Myrmicine ant | No | Yes; Dendrobatidae (Oophaga) | Daly et al 2005, "Alkaloids:  Chemical and Biological Perspectives" 1999 |
| 211A DHQ | Myrmicine ant | No | Yes; Dendrobatidae (Oophaga) | Daly et al 2005, "Alkaloids:  Chemical and Biological Perspectives" 1999 |
| 211K DHQ | Myrmicine ant | No | Yes; Dendrobatidae (Oophaga) | Daly et al 2005, "Alkaloids:  Chemical and Biological Perspectives" 1999 |
| 211L 5,6,8-I | Unknown | No | Yes, Dendrobatidae | Daly et al 2005 |
| 223E Izidine 1st Isomer | Unknown | Yes | Yes; Dendrobatidae (Oophaga) | Daly et al 2005, Saporito et al 2007, "Alkaloids:  Chemical and Biological Perspectives" 1999 |
| 225D 5, 8-I 2ndIsomer | Mite | No | Yes; Dendrobatidae (Oophaga) | Daly et al 2005, "Alkaloids:  Chemical and Biological Perspectives" 1999, Saporito et al 2012 |
| 225K 5,6,8-I | Mite | Yes | Yes; Dendrobatidae (Oophaga) | Daly et al 2005; Bolton 2017, Saporito et al 2012 |
| 233B Izidine | Unknown | No | Yes; Dendrobatidae | Daly et al 2005, "Alkaloids:  Chemical and Biological Perspectives" 1999 |
| 235M Tricyclic | Unknown | Yes; betsileo | Yes; Dendrobatidae | Daly et al 2005, "Alkaloids:  Chemical and Biological Perspectives" 1999, Daly et al 1996 |
| 235T Tricyclic 1st isomer | Unknown | Yes | No | Daly et al 2005 |
| 237E 3,5-I | Ant | No | Yes | Daly et al 2005, "Alkaloids:  Chemical and Biological Perspectives" 1999, Saporito et al 2012 |
| 237O Tricyclic | Unknown | No | Yes, Bufonidae | Daly et al 2005 |
| 239K 3,5-P | Ant | Yes; aurantiaca, milotymp | No | Daly et al 2005, Garrafo et al 1993, "Alkaloids:  Chemical and Biological Perspectives" 1999, Saporito et al 2012 |
| 239W 5,6,8-I | Unknown | Yes | No | Daly et al 2005 |
| 243C 5, 8-I | Mite | Yes; baroni | Yes; Dendrobatidae (Oophaga) | Daly et al 2005, "Alkaloids:  Chemical and Biological Perspectives" 1999, Daly et al 1996, Bolton 2017, Saporito et al 2012 |
| 247C 3,5-I | Ant | Yes; betsileo, laevigata | No | Daly et al 2005, "Alkaloids:  Chemical and Biological Perspectives" 1999, Daly et al 1996, Saporito et al 2012 |
| 249A 3,5-I 1st Isomer | Ant | Yes; aurantiaca, baroni | Yes; Dendrobatidae (Oophaga) | Daly et al 2005, Daly et al 1996, "Alkaloids:  Chemical and Biological Perspectives" 1999, Saporito et al 2012 |
| 249X 3,5-P | Unknown | Yes | No | Daly et al 2005 |
| 251O 3,5-P | Ant: Tetramorium electrum | Yes; madagascarensis, baroni | No | Daly et al 2005, "Alkaloids:  Chemical and Biological Perspectives" 1999, Clark et al 2005, Saporito et al 2012 |
| 251Q Izidine | Unknown | Yes; betsileo | No | Daly et al 2005, Daly et al 2006, "Alkaloids:  Chemical and Biological Perspectives" 1999 |
| 251U 5, 8-I | Mite | Yes | Yes; Dendrobatidae (Oophaga) | Daly et al 2005, "Alkaloids:  Chemical and Biological Perspectives" 1999, Saporito et al 2012, Bolton 2017 |
| 253B 5, 8-I 1stIsomer | Mite | Yes; aurantiaca, milotymp | Yes; Dendrobatidae (Oophaga) | Daly et al 2005, "Alkaloids:  Chemical and Biological Perspectives" 1999, Saporito et al 2012, Bolton 2017 |
| 253F PTX | Unknown | Yes | Yes; Dendrobatidae (Oophaga) | Daly et al 2005, "Alkaloids:  Chemical and Biological Perspectives" 1999, Saporito et al 2007 |
| 267W 5,6,8-I | Unknown | Yes | No | Daly et al 2005 |
| 275C 3,5-I 1st Isomer | Ant | Yes; baroni, expectata, laevigata, pulchra | Yes; Dendrobatidae (Oophaga) | Daly et al 2005, Daly et al 1996, Saporito et al 2007, "Alkaloids:  Chemical and Biological Perspectives" 1999 |
| 275E 5,6,8-I 2nd Isomer | Mite | Yes | Yes; Dendrobatidae (Oophaga) | Daly et al 2005, Saporito et al 2007, |
| 277I Izidine | Unknown | Yes | No | Daly et al 2005 |
| 291E DeoxyPTX 1st Isomer | Unknown | Yes; baroni, betsileo, laevigata, pulchra | No | Daly et al 2005, Daly et al 1996, "Alkaloids:  Chemical and Biological Perspectives" 1999 |
| 305A aPTX | Mite | Yes; aurantiaca, crocea | Yes; Dendrobatidae (Oophaga) | Daly et al 2007, Garrafo et al 1993, Saporito et al 2007, "Alkaloids:  Chemical and Biological Perspectives" 1999 |
| 307A PTX (Pumiliotoxin A) minorIsomer | Ants; Formicine, Brachymyrmex, Paratrechina | Yes; aurantiaca, milotymp | Yes; Dendrobates, Dendrobatidae (Oophaga) | Clark 2005, Daly et al 2005, Garrafo et al 1993, "Alkaloids:  Chemical and Biological Perspectives" 1999, Saporito et al 2004 |
| 307G PTX | Unknown | Yes; aurantiaca, betsileo, cowanii, laevigata, pulchra, viridis, | Yes | Daly et al 1996, Daly et al 2005, "Alkaloids:  Chemical and Biological Perspectives" 1999 |
| 321B hPTX | Unknown | Yes; betsileo, laevigata, pulchra | Yes; Bufonidae, Dendrobatidae | Daly et al 2005, Daly et al 1996, Garrafo et al 1993, "Alkaloids:  Chemical and Biological Perspectives" 1999 |
| 321D hPTX 1st Isomer | Unknown | Yes | No | Daly et al 2005 |
| 323A PTX (Pumiliotoxin B) | Mite, Ants; Formicine, Brachymyrmex, Paratrechina | Yes; aurantiaca, milotymp | Yes; Dendrobatidae (Oophaga), Bufonidae, Myobatrachidae | Clark 2005, Daly et al 2005, Garrafo et al 1993, "Alkaloids:  Chemical and Biological Perspectives" 1999, Saporito et al 2006, Bolton 2017 |
| 337B hPTX | Unknown | Yes; aurantiaca, baroni, betsileo, pulchra | No | Daly et al 2005, Daly et al 1996, "Alkaloids:  Chemical and Biological Perspectives" 1999 |
| 380 DHQ-dimer | Unknown | No | Yes; Dendrobatidae | Daly et al 2005 |
